# Radical SAM Enzymes and Ribosomally‐Synthesized and Post‐translationally Modified Peptides: A Growing Importance in the Microbiomes

**DOI:** 10.3389/fchem.2021.678068

**Published:** 2021-07-19

**Authors:** Alhosna Benjdia, Olivier Berteau

**Affiliations:** Université Paris-Saclay, INRAE, AgroParisTech, Micalis Institute, ChemSyBio, Jouy-en-Josas, France

**Keywords:** RiPPs, antibiotic, radical SAM enzyme, peptide, natural product (bio)synthesis, radical AdoMet enzyme, microbiome, microbiota

## Abstract

To face the current antibiotic resistance crisis, novel strategies are urgently required. Indeed, in the last 30 years, despite considerable efforts involving notably high-throughput screening and combinatorial libraries, only few antibiotics have been launched to the market. Natural products have markedly contributed to the discovery of novel antibiotics, chemistry and drug leads, with more than half anti-infective and anticancer drugs approved by the FDA being of natural origin or inspired by natural products. Among them, thanks to their modular structure and simple biosynthetic logic, ribosomally synthesized and posttranslationally modified peptides (RiPPs) are promising scaffolds. In addition, recent studies have highlighted the pivotal role of RiPPs in the human microbiota which remains an untapped source of natural products. In this review, we report on recent developments in radical SAM enzymology and how these unique biocatalysts have been shown to install complex and sometimes unprecedented posttranslational modifications in RiPPs with a special focus on microbiome derived enzymes.

Despite being known since the dawn of microbiology, it is only recently that various secondary metabolites derived from peptide precursors have been recognized as a unified family of natural products now called ribosomally synthesized and posttranslationally modified peptides (RiPPs) (Arnison et al., 2013; Montalban-Lopez et al., 2021). RiPPs such as nisin have been first investigated because of their antibiotic properties (Mattick and Hirsch, 1947); however, with the growing recognition that in the environment, bacteria live in complex communities rather than as free planktonic cells (Hall-Stoodley et al., 2004; Bjarnsholt et al., 2013), the functions of RiPPs are likely much more diverse. Indeed, bacteria can adopt complex lifestyles (Hibbing et al., 2010), with RiPPs providing not only a competitive advantage against competing species (Telhig et al., 2020), but also playing a major role in communication (Ibrahim et al., 2007; Liu et al., 2010), biofilm formation and the acquisition of metals (Hider and Kong, 2010; Dassama et al., 2017). More recently, RiPPs have also been proposed to play a pivotal role in the homeostasis of the human microbiota (Benjdia and Berteau, 2016; Balskus, 2018; Chittim et al., 2018; Balty et al., 2020), with the recent discovery of several novel antibiotics from this complex ecosystem (Rothschild et al., 2018) including colicin V (Cohen et al., 2018), humimycin (Chu et al., 2018) and ruminococcin C (Balty et al., 2019; Chiumento et al., 2019; Balty et al., 2020).

While the biosynthesis of RiPPs follows a common logic with the translation of a precursor peptide containing a leader (or a follower) sequence, the installation of posttranslational modifications and the cleavage of the leader peptide (Figures 1A,B) (Arnison et al., 2013; Ortega and van der Donk, 2016; Funk and van der Donk, 2017; Montalban-Lopez et al., 2021), RiPPs display an extraordinary structural diversity largely due to the various posttranslational modifications installed by diverse and unrelated enzymes (Montalban-Lopez et al., 2021). In the last decade, radical SAM enzymes have emerged arguably as the most versatile biocatalysts catalyzing an unsurpassed diversity of post-translational modifications in RiPPs (Vey and Drennan, 2011; Broderick et al., 2014; Benjdia and Berteau, 2016). For instance, they have been shown to install chemically unrelated posttranslational modifications including thioether (Flühe, 2012; Benjdia et al., 2016) and carbon–carbon (Schramma et al., 2015; Barr et al., 2016; Benjdia et al., 2017b) bonds, unusual *C*-methylation (Freeman et al., 2012; Huo et al., 2012; Pierre et al., 2012; Benjdia et al., 2015; Parent et al., 2016; Freeman et al., 2017), and epimerization (Freeman et al., 2012; Benjdia et al., 2017c; Parent et al., 2018) (Figure 1C).

**FIGURE 1:**
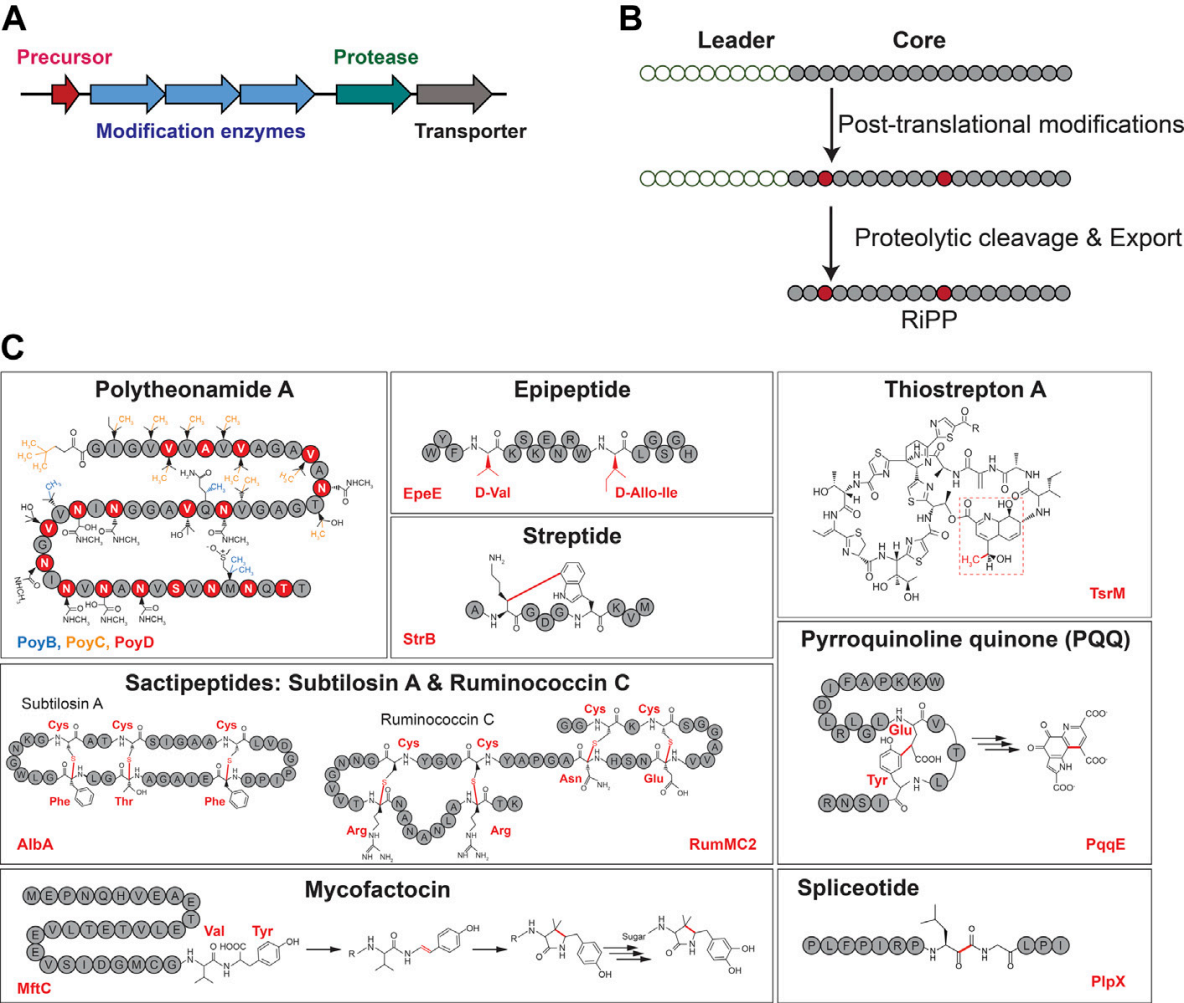
Generic RiPP biosynthetic gene cluster **(A)** and biosynthetic logic **(B)**. **(C)** Representative examples of RiPPs with posttranslational modifications installed by radical SAM enzymes. Radical SAM enzyme names are indicated at the bottom of each panel.

In this review, we will cover recent advances in our understanding of radical SAM enzymes involved in RiPP biosynthesis from a mechanistic to a structural perspective, with a special emphasis on radical SAM enzymes involved in the formation of thioether bond, epimerization, carbon–carbon and carbon–oxygen bonds for which major progresses on their mechanisms, structures, and biological functions have been accomplished in the last years. In addition, these enzymes and their metabolites have been shown to play important functions in various microbiomes from antibiotic activity to quorum sensing (Benjdia and Berteau, 2016; Li and Rebuffat, 2020).

## Radical SAM Enzymes: An Emerging Superfamily of Enzymes

Radical SAM enzymes were recognized 20 years ago as an emerging superfamily of enzymes (Sofia et al., 2001). According to the Structure–Function Linkage Database (http://sfld.rbvi.ucsf.edu/), these enzymes form the most diverse and large superfamily of enzymes with more than 500,000 predicted members (radicalsam.org) and ∼100 distinct families. However, this superfamily has one of the lowest numbers of available structures (∼20 known structures), and more than half of its different subgroups have no predicted function, underscoring the poor knowledge we still have on these biocatalysts. Finally, this superfamily is still expanding with novel reactions and radical SAM enzymes regularly reported.

As a general feature, radical SAM enzymes are metalloenzymes characterized by an [4Fe-4S]^2+/1+^ cluster coordinating an organic cofactor: *S*-adenosyl-L-methionine (SAM). They have been shown to use this cofactor to catalyze various radical-based reactions; however, only a handful have been characterized in detail. Thanks to the powerful radical activation of their substrates, radical SAM enzymes have been shown to catalyze complex and often critical transformations in the biosynthetic pathways of natural compounds or in the central metabolism (Broderick et al., 2014; Benjdia and Berteau, 2016; Berteau, 2018). For instance, they have been reported to be involved in key biological processes such as DNA repair (Rebeil et al., 1998; Chandor et al., 2006; Friedel et al., 2006; Benjdia et al., 2012), protein posttranslational modification (Ollagnier et al., 1996; Berteau et al., 2006; Benjdia et al., 2007a; Benjdia et al., 2007b; Benjdia et al., 2008; Arragain et al., 2009; Benjdia et al., 2009; Benjdia et al., 2010), nucleic acid modification (Pierrel et al., 2003; Grove et al., 2011) and the biosynthesis of cofactors (Layer et al., 2006; Decamps et al., 2012; Harmer et al., 2014; Philmus et al., 2015) and vitamins (Vander Horn et al., 1993; Sanyal et al., 1994; Kriek et al., 2007; Chatterjee et al., 2008). More recently, there has been a tremendous increase in the discovery of radical SAM enzymes involved in RiPP biosynthetic pathways (Wang and Frey, 2007; Fluhe et al., 2012; Pierre et al., 2012; Allen and Wang, 2014; Benjdia et al., 2015; Benjdia et al., 2017a; Parent et al., 2018; Balty et al., 2019; Imai et al., 2019; Balty et al., 2020).

**FIGURE 2:**
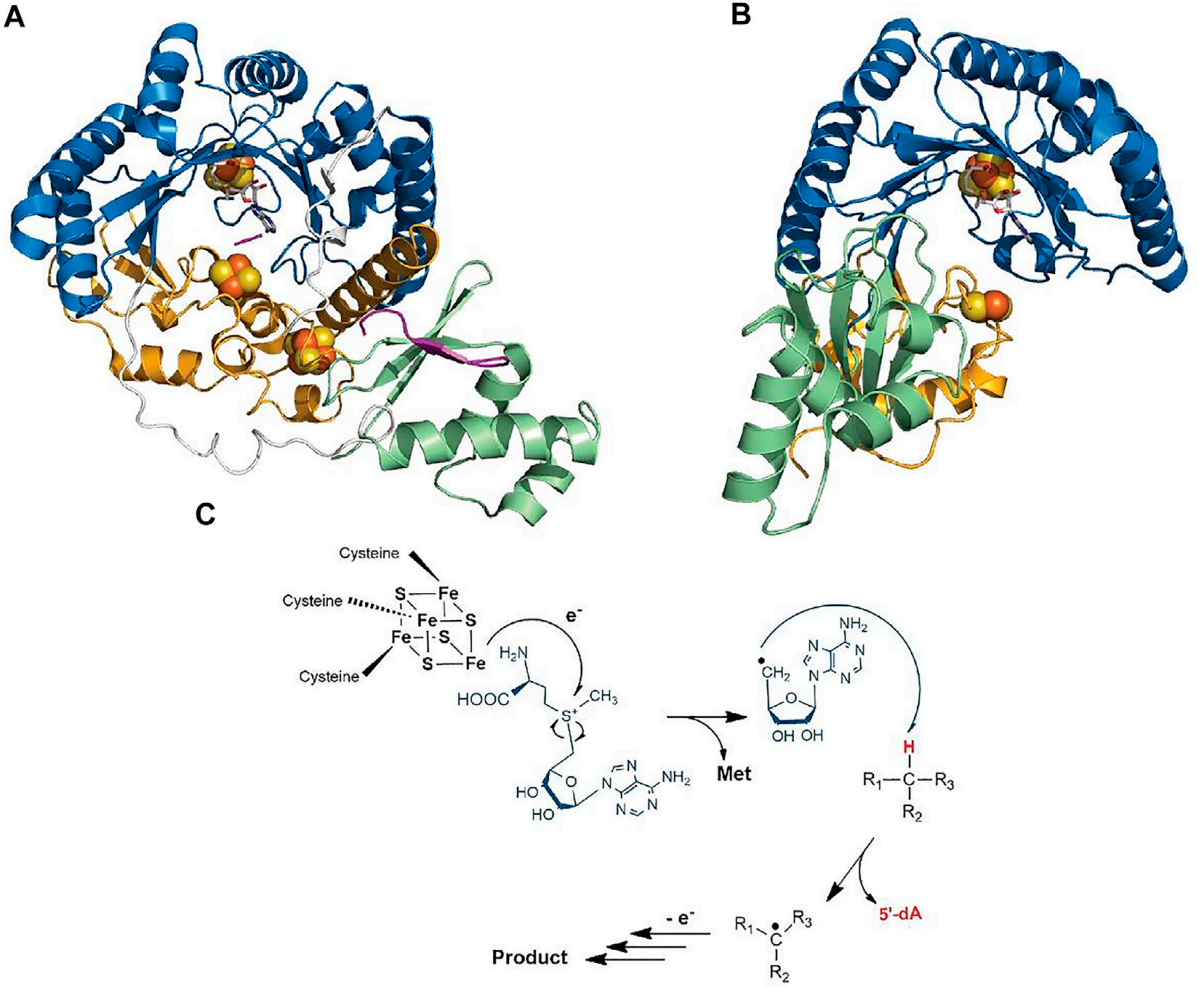
**C**rystal structure of RiPP-modifying radical SAM enzymes and generic mechanism of radical SAM enzymes. **(A)** Structure of CteB (Grove et al., 2017) and **(B)** SkfB (Grell et al., 2018). The radical SAM domain is shown in blue with the active site [4Fe-4S]^2+^ cluster coordinating the SAM cofactor which is colored in white. The N-terminal RRE domain is depicted in green, and the C-terminal SPASM (in CteB) or twitch domain (in SkfB) is shown in orange. **(C)** Generic mechanism of radical SAM enzymes: One-electron reduction of the radical SAM cluster leads to the homolytic cleavage of SAM. The 5′-dA• radical formed abstracts a substrate H-atom leading to the formation of a radical intermediate. After rearrangement, the product is released. Although used by most known radical SAM enzymes, variations of this mechanism are known (Zhang et al., 2010; Benjdia et al., 2015; Rohac et al., 2016; Joshi et al., 2021).

Despite catalyzing such diverse reactions, radical SAM enzymes share common structural and mechanistic features. Briefly, the general mechanism of these enzymes can be described as follows: a molecule of SAM localized at the top of a conserved TIM-barrel fold [(β/α)_6_ or (β/α)_8_ fold] (Vey and Drennan, 2011) (Figures 2A,B) interacts with the catalytic [4Fe-4S]^2+/1+^ cluster of the enzyme. In a unique manner, an electron transfer from the reduced [4Fe-4S]^1+^ cluster to SAM induces its homolytic cleavage. A highly reactive species, the 5′-deoxyadenosyl radical, is then produced, which in turn initiates the radical reaction through, in most cases, the activation of a C–H bond. Following H-atom abstraction, a radical substrate intermediate is formed and undergoes specific rearrangements concluding the reaction (Frey et al., 2008; Broderick et al., 2014). The process leading to the release of the product is likely assisted by the protein matrix which not only positions the reactants but also controls the outcome of the reaction (Figure 2C) (Benjdia et al., 2012).

Interestingly, in addition to the radical SAM domain, many radical SAM enzymes possess additional domains and cofactors. Notably, a large proportion of the radical SAM enzymes involved in RiPP biosynthesis harbor the so-called RRE (peptide recognition element) (Burkhart et al., 2015) and a SPASM or a twitch domain (Haft and Basu, 2011; Goldman et al., 2013a). The RRE domain, which is generally located in the N-terminal part of the enzyme, is not restricted to radical SAM enzymes but widespread among RiPP-modifying enzymes where its function is to interact with the leader peptide. Its overall structure is generally well conserved and consists of a winged Helix-Turn-Helix (wTHT) fold characterized by three-stranded antiparallel β-sheets and three α-helices (Figures 2A,B) (Burkhart et al., 2015). In contrast, the SPASM/twitch domain is located only in the *C*-terminal part of radical SAM enzymes and characterized by the presence of one or two iron–sulfur clusters (Figures 2A,B) whose function remains controversial. In the last five years, a new picture has emerged regarding mechanistic evolution within the radical SAM enzyme superfamily and how they have evolved to catalyze RiPP post-translational modifications.

### Thioether Bonds

**FIGURE 3:**
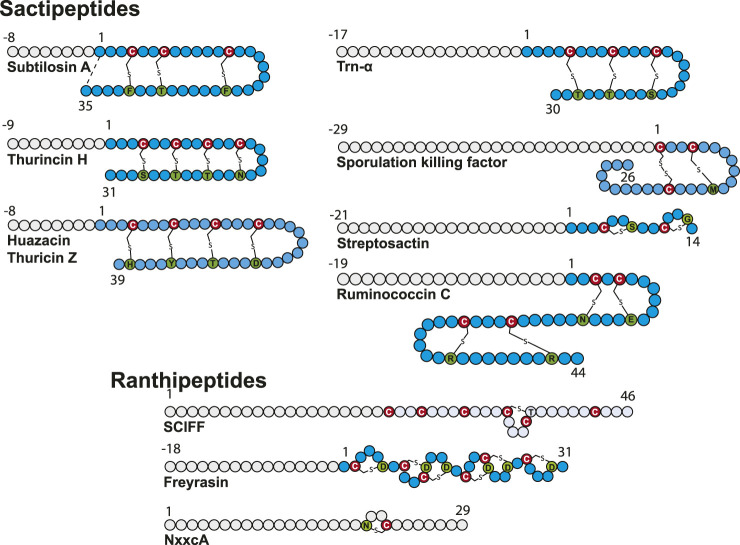
Structure of sactipeptides and ranthipeptides. Circles in gray and blue represent amino acid from the leader peptide and the core sequence, respectively. The donor cysteine residues are indicated by a red circle, and acceptor amino acid residues are depicted in green.

Thioether bonds were among the first posttranslational modifications identified in RiPPs. Indeed, the presence of the so-called lanthionine and methyl-lanthionine bridges (linking a cysteine residue to a serine or a threonine residue) was reported several decades ago and delineates the lanthipeptide family (Arnison et al., 2013), with nisin being the prototype of this family. These bridges are formed by a variant of the well-known Michael addition (i.e., nucleophile addition to an α, β unsaturated carbonyl group), leading to the formation of C_β_–thioether bridges. Later, subtilosin A, a macrocyclic peptide antibiotic active against *Listeria monocytogenes* (Zheng et al., 2000) (Figures 1C, 3), was uncovered in *B. subtilis*. Characterization of its biosynthetic cluster revealed the presence of a radical SAM enzyme (AlbA) playing a key role for its biosynthesis (Zheng et al., 2000). Subsequently, the structure of subtilosin A was solved and shown to contain, in addition to a head-to-tail cyclization, three unusual thioether bonds connecting three cysteine residues to three C_α_ atoms, defining a novel family of bacteriocins (Kawulka et al., 2004). Soon after, another peptide with this unusual linkage was also discovered in *B. subtilis* and proved to play a key role in the transition from planktonic cells to spores (Gonzalez-Pastor et al., 2003; Liu et al., 2010). This cannibalistic factor was named sporulation killing factor (SkfA), and its biosynthesis was shown to be under the dependence of another radical SAM enzyme called SkfB (Gonzalez-Pastor et al., 2003; Fluhe et al., 2013). Contrary to subtilosin A, SkfA contains a single thioether bond and a disulfide bridge as head-to-tail cyclization. Adding to this growing family of unusual thioether peptides, thurincin H (Sit et al., 2011) and thuricin CD (Trn-α and Trn-β) (Rea et al., 2010), both produced by *Bacillus thuringiensis*, were later reported as containing four and three C_α_–thioether bridges, respectively (Figure 3).

Biosynthesis of these RiPPs, containing sulfur-to-α-carbon thioether bonds involving a donor cysteine and an acceptor amino acid residue, is under the strict dependence of radical SAM enzymes. First dubbed sactibiotics (Murphy et al., 2011), they are now collectively named sactipeptides (Arnison et al., 2013). In addition to hyicin, a RiPP highly homologous to subtilosin A identified in *Staphylococcus hyicus* (Duarte et al., 2018), ruminococcin C (Balty et al., 2019; Balty et al., 2020), thuricin Z (Hudson et al., 2019; Mo et al., 2019), and streptosactin (Bushin et al., 2020) were recently discovered and their biosynthetic pathway deciphered, bringing the number of known sactipeptides to seven.

Sactipeptide precursors contain a leader sequence of 8–29 amino acid residues and a core sequence ranging from 26 for SkfA (Gonzalez-Pastor et al., 2003) to 44 amino acid residues for ruminococcin C (Balty et al., 2019). Except for ruminococcin C (Balty et al., 2019) and streptosactin (Bushin et al., 2020), all known sactipeptides possess a hairpin structure with all donor cysteine residues from the N-terminal part linked to acceptor amino acid residues from the C-terminal part (Figure 3). The number of thioether linkages is generally between 1 and 4, and they involve all the major groups of amino acid residues: negatively (Glu and Asp) and positively (Lys, Arg and His) charged residues, residues with polar side chains (Ser, Thr and Asn), hydrophobic and aromatic residues (Met, Tyr and Phe) and glycine. In line with their distinct architecture, only ruminococcin C and streptosactin do not originate from *Bacillus* species. Indeed, both RiPPs have been isolated from members of the human microbiota (*Ruminococcus gnavus*) or food bacteria (*Streptococcus thermophilus*).

### Biological Function of Sactipeptides

Subtilosin A was isolated due to its antimicrobial activity against several Gram-positive bacteria, including the food-borne pathogen *Listeria monocytogenes* (Babasaki et al., 1985; Zheng et al., 2000). Only recently, information about its mode of action has been uncovered. Subtilosin A appears to perturb the *L. monocytogenes* lipid bilayer, resulting in intracellular damages (van Kuijk et al., 2012). SkfA on the other hand is a cannibalistic factor playing a key role in the transition from planktonic cells to spores (Gonzalez-Pastor et al., 2003). Other sactipeptides exert antimicrobial activity against Gram-positive bacteria with sometimes a narrow spectrum of action. Targeted species include *Clostridium* species (thuricin CD and ruminococcin C), *L. monocytogenes* (thurincin H), *Bacillus cereus* (thuricin Z) and *S. thermophilus* (streptosactin). Whether their mode of action is similar to the one of subtilosin A remains to be fully elucidated.

### Mechanism for Cα–Thioether Bond Formation

**FIGURE 4:**
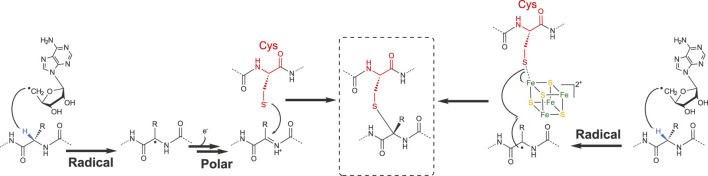
Radical and polar alternatives for the formation of C_α_–thioether bridges.

First *in vitro* studies regarding the formation of thioether bonds by radical SAM enzymes were published almost a decade ago (Flühe, 2012; Fluhe et al., 2013). Not surprisingly, these studies addressed the mechanism of AlbA and SkfB involved in subtilosin A and SkfA biosynthesis, respectively. These studies supported that C_α_-thioether bond formation was under the dependence of the leader peptide and radical SAM enzymes containing two [4Fe-4S] clusters (Flühe, 2012; Fluhe et al., 2013). Later, it was shown that AlbA in fact contains three [4Fe-4S] clusters (Benjdia et al., 2016), and SkfB one [4Fe-4S] and one [2Fe-2S] clusters (Grell et al., 2018). By using rationally designed synthetic peptide substrates and labeling experiments, Benjdia and coworkers probed the AlbA mechanism in detail (Benjdia et al., 2016). One synthetic peptide, in which the residues 27–35 were covalently linked to residues 1–6 by an amide bond, proved to be an efficient substrate and amenable to labeling experiment. Activity of AlbA on this synthetic peptide demonstrated that 1) its activity is not strictly dependent on the leader peptide and 2) the C_α_ H-atom of the acceptor amino acid residue is the target of the 5′-dA radical generated after SAM cleavage (Benjdia et al., 2016). Following this study, Bandarian and coworkers took advantage of the substrate promiscuity of SkfB to demonstrate that like AlbA, SkfB catalyzes H-atom abstraction on the C_α_ H-atom (Bruender and Bandarian, 2016). Indeed, SkfA contains a single thioether bond involving Met12 as an acceptor residue which can be substituted by an Ala residue without affecting SkfB activity. By using a peptide substrate containing either a perdeuterated alanine residue or an alanine labeled on the β-methyl moiety, it was possible to establish that only the C_α_ H-atom is abstracted during catalysis. This strategy was successfully applied to investigate the formation of thioether bonds in ruminococcin C (Balty et al., 2020) and streptosactin (Bushin et al., 2020). Based on these studies, two mechanisms for thioether bond formation by radical SAM enzymes are currently proposed (Figure 4). Following SAM cleavage, the 5′-dA radical abstracts a C_α_ H-atom on the acceptor amino acid residue, a process thermodynamically favorable considering the C_α_ H-atom bond dissociation energy. The carbon-centered radical intermediate formed could then either react with the donor cysteine residue coordinated to an auxiliary iron–sulfur cluster (Flühe, 2012) or form a ketoimine intermediate which can be readily trapped by the nucleophilic thiolate group of the donor cysteine residue (Benjdia et al., 2016). Interestingly, a recent study on ruminococcin C, combining the investigation of mutated and labeled peptides, has shown that during LC-MS fragmentation, instead of α, β-dehydro-amino acid, ketoimine intermediates are likely formed, supporting the intermediacy of such species in C_α_–thioether bond chemistry (Balty et al., 2020).

As described above, the wide majority of sactipeptides contain several thioether bridges. The initial study on AlbA suggested a rapid conversion of the singly- and doubly bridged species into subtilosin A; however, it was unclear if this was the result of a processive mode of action (Flühe, 2012). Studies on ruminococcin C clearly established that thioether bond formation occurs as an orderly process, with the internal bond (according to the hairpin structure) being formed before the second bond with a strict *N*-to-*C* directionality either *in vitro* or *in vivo* (Balty et al., 2019; Balty et al., 2020). In contrast, the formation of the thioether bonds in streptosactin follows a *C*-to-*N* directionality (Bushin et al., 2020). These studies strongly support that at least for some sactipeptides, thioether bond formation is likely a processive rather than an associative process.

### Novel Thioether Bonds

While most of the radical SAM enzymes involved in RiPP biosynthesis investigated to date have been shown to target C_α_ atoms, it has been recently demonstrated that the diversity of thioether bonds is greater than anticipated. Indeed, radical SAM enzymes are also able to catalyze formation of C_β_ (Caruso et al., 2019a) and C_γ_ (Hudson et al., 2019) thioether bonds. These RiPPs called now Ranthipeptides: **ra**dical **n**on-α **thi**oether peptides encompass three SCIFFs (Haft and Basu, 2011; Grove et al., 2017; Hudson et al., 2019) and NxxcA (Caruso et al., 2019a) (Figure 3). The SCIFFs (**s**ix **c**ysteines **i**n **f**orty **f**ive residues) were shown to be widely spread among *Clostridia* (Haft and Basu, 2011), arguing against a role of antimicrobial compound. The first SCIFF characterized *in vitro*, Tte1186a, originated from *Caldanaerobacter subterraneus*. Despite the presence of six cysteine residues, only one thioether bond between Cys32 (i.e., the fourth cysteine in the precursor peptide) and Thr37 has been identified (Bruender et al., 2016). Biochemical characterization of another SCIFF from *Clostridium thermocellum* (CteA) showed the formation of a similar bond, linking again Cys32 and Thr37 located five residues apart (Grove et al., 2017). Initially, it was hypothesized that SCIFFs contain C_α_–thioether bonds. However, Mitchell and coworkers, while investigating several putative sactipeptide operons including SCIFF peptides, observed that 1) peptide products (Tte1186 and CteA) do not undergo the characteristic mass fragmentation for sactionine linkages and that 2) SCIFF maturases exhibit significant sequence similarities with a non-RiPP radical SAM enzyme QhpD, catalyzing S-C_β_ (Cys-Asp) and S-C_γ_ (Cys-Glu) thioether bond formation in quinohemoprotein amine dehydrogenase (QHNDH) (Ono et al., 2006). Recombinant expression of CteA with its maturase CteB in *E. coli* led to the formation of a RiPP with a unique thioether bond linking Cys32 to a threonine residue (Thr34) (Figure 3) located two positions away from Thr37, giving rise to a shorter cycle (Hudson et al., 2019). The nature of these discrepancies is unclear and will need to be clarified; however, thorough analysis showed that, instead of C_α_-thioether bond, this SCIFF contains a C_γ_–thioether bond. Open questions remain regarding the nature of the active form of this SCIFF and whether or not other donor cysteine residues from the precursor peptide are engaged in thioether bonds. The other known ranthipeptides are the SCIFF from *Paenibacillus polymyxa* called freyrasin and NxxcA from *Streptococcus orisratti* (Caruso et al., 2019a; Hudson et al., 2019). While the latter RiPP contains a single thioether bridge connecting Cys23 to Asn20, freyrasin harbors six thioether bridges connecting cysteine to aspartate residues (Figure 3). Interestingly, NMR analysis showed that both NxxcA and the SCIFF freyrasin contain C_β_–thioether bridges (Caruso et al., 2019a; Hudson et al., 2019).

**FIGURE 5:**
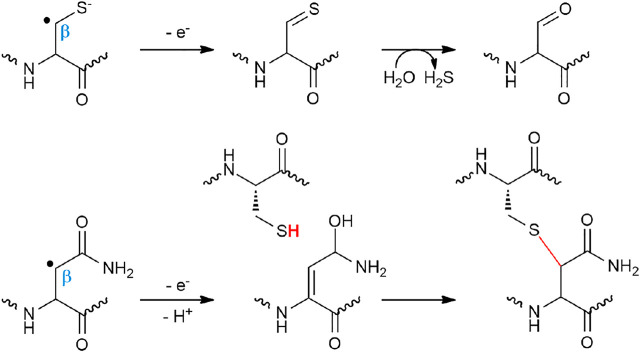
Proposed mechanisms for anSME and NxxcB catalyzing cysteine oxidation and C_β_–thioether bond formation, respectively.

These radical reactions involving a C_β_ or C_γ_ atoms are reminiscent of anSME, a radical SAM enzyme involved in sulfatase maturation (Benjdia et al., 2007b). This enzyme converts an active-site Ser or Cys residue into a C_α_-formylglycine (Dierks et al., 1997; Berteau et al., 2006; Benjdia et al., 2007a; Benjdia et al., 2007b; Benjdia et al., 2009) by targeting the C_β_ Η-atom like in NxxcA (Caruso et al., 2019a) and freyrasin (Hudson et al., 2019). However, the fate of the radical intermediate differs with the formation of thioether bonds in ranthipeptides (Figure 5). In NxxcA, it was proposed that following C_β_ H-atom abstraction, deprotonation of the remaining Asn20 C_α_ H-atom gives rise to an α, β-insaturation which could be resolved by electrophilic addition of the thiolate group of Cys23 (Caruso et al., 2019a). However, C_α_ deprotonation is a chemically unfavorable process. Alternatively, by analogy to the mechanism proposed for AlbA and SkfB (Flühe, 2012; Fluhe et al., 2013), the cysteine residue of NxxcA (Cys23) could interact with an auxiliary [4Fe-4S] cluster located in the SPASM domain (Caruso et al., 2019a) and reacts with the radical peptide intermediate to form a thioether bond. Intriguingly, in SCIFFs (Hudson et al., 2019; Precord et al., 2019) and NxxcA (Caruso et al., 2019a), the carbon atom of the acceptor residue (i.e., Asn, Thr and Asp), involved in thioether bond, is located downstream a functional group (i.e., amide, hydroxyl or acid group). This opens the possibility that once the carbon-centered radical is formed, interconversion would lead to the formation of a dehydro-amino acid residue which could directly react with the thiolate group of the donor cysteine residue.

### Structures of Sactisynthases and Ranthisynthases

Structures of radical SAM enzymes covering distinct chemistries are available, but very little structural information are reported regarding RiPP-modifying enzymes (Benjdia et al., 2017a; Mahanta et al., 2017a). To date, the structure of only one sactisynthase, SkfB, is available (Figure 2B) (Grell et al., 2018). Although initial studies indicated this enzyme to contain two [4Fe-4S] clusters, the crystal structure revealed the presence of a [2Fe-2S] cluster, in addition to the radical SAM [4Fe-4S] cluster. Interestingly, the [2Fe-2S] cluster is coordinated by only three cysteine residues, leaving an open coordination to bind the substrate. However, currently one cannot exclude that the remote Cys333 located in the vicinity of the [2Fe-2S] cluster could serve as a ligand or being involved in interconversion to a [4Fe-4S] cluster. The structure of the ranthisynthase CteB (Grove et al., 2017), despite the presence of two [4Fe-4S] clusters in the *C-*terminal SPASM domain, provided a similar picture, with one cluster having an open coordination (Figure 2A). In addition, the “free iron” from this cluster is oriented toward the entrance of the active site and properly placed to interact with the incoming peptide substrate. Supporting this function, the structure of CteB bound to a peptide, encompassing residues 1–21 from the CteA (corresponding to the leader peptide sequence and the first three residues of the core peptide), revealed that Cys21, despite being not involved in a thioether bond, could interact with the free iron of the SPASM-domain [4Fe-4S] cluster (Grove et al., 2017). It was thus proposed that this peptide mimic the interaction between the actual donor residue (i.e., Cys32) and the auxiliary [4Fe-4S] cluster (Grove et al., 2017). Although none of the above structures were solved in the presence of the core sequence where posttranslational modifications take place, both structures support that clusters from the SPASM/twitch domain, in addition to having a role in the redox chemistry, could also play a role in substrate binding and activation. Moreover, the structures of SkfB (Grell et al., 2018) and CteB (Grove et al., 2017) also share the presence of an RRE in their *N*-terminal part. Although no peptide ligand was present in SkfB, in CteB, it was shown that the leader peptide from CteA adopts a β-strand conformation sitting at the interface of α3-helix and β3-sheet of the RRE domain, as expected (Grove et al., 2017).

### Epimerization

Sactisynthases are not the only radical SAM enzymes targeting the C_α_ atom of RiPPs. Recently, another family of enzymes has been shown to catalyze Cα H-atom abstraction, the radical SAM epimerases. Radical SAM enzymes were predicted to catalyze epimerization reactions more than a decade ago, following the investigation of the avilamycin A biosynthetic pathway (Boll et al., 2006). Indeed, *in vivo* studies showed that a radical SAM enzyme was likely responsible for a critical C2 epimerization in the biosynthesis of this antibiotic. In 2012, the first peptide radical SAM epimerase, PoyD, was identified in the biosynthetic pathways of polytheonamide A, a hyper-modified and cytotoxic RiPP isolated from the sponge *Theonella swinhoei* (Freeman et al., 2012). Although epimerization was known in other RiPPs such as lanthipeptides which contain D-Ala and D-2-aminobutyrate following dehydration of Ser and Thr residues (Cotter et al., 2005; Lohans et al., 2014; Huo and van der Donk, 2016), it was the first time that direct amino acid epimerization in a RiPP was shown. Among the 48 posttranslational modifications introduced in polytheonamide A, PoyD was proposed to catalyze no less than 18 epimerizations on the precursor peptide PoyA. This peptide belongs to the family of proteusins characterized by the presence of a large *N*-terminal leader region sharing similarities to the α-subunit of nitrile hydratases. *In vivo* and *in vitro* studies on PoyD revealed unprecedented directional epimerizations from the *C*-terminal to the *N*-terminal part with a strict pattern of epimerization every 1,3-position (Morinaka et al., 2017; Parent et al., 2018). Interestingly, PoyD is also able to generate *in vitro* various epimerized products with natural and unnatural epimerization patterns (Parent et al., 2018). Furthermore, the fact that PoyD is able to install posttranslational modifications in a short synthetic peptide encompassing only the first eight amino acid residues of the core peptide strongly suggested that the enzyme activity is leader peptide independent (Parent et al., 2018).

**FIGURE 6:**
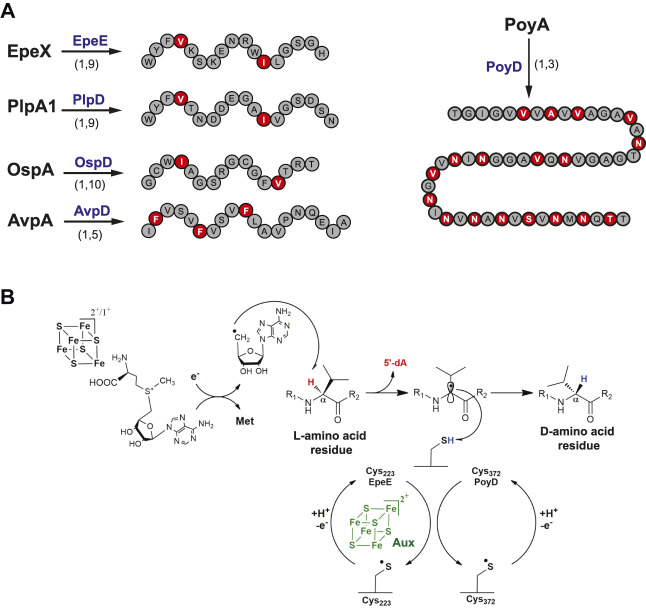
Epipeptides and proteusins. **(A)** Epimerization pattern of proteusins and epipeptide. Above the arrows, names of the radical SAM enzymes catalyzing epimerization reactions. Below the arrows, the numbers indicate the epimerization patterns. **(B)** Proposed mechanism for epimerization catalyzed by radical SAM epimerases.

Genome mining has been used to uncover new biosynthetic gene clusters (BGCs) containing PoyD homologues and putative precursor peptides. Several BGCs were identified in cyanobacteria, and novel RiPP epimerases (i.e., PlpD, OspD, and AvpD) were characterized (Morinaka et al., 2014; Freeman et al., 2017; Vagstad et al., 2019). These novel RiPPs, despite harboring a limited number of epimerizations compared to polytheonamide A, are all collectively called proteusins (Figure 6A). Soon after, in *Bacillus subtilis*, the investigation of a gene cluster known to induce the expression of LiaRS (Butcher et al., 2007), a major component of the bacterial cell envelope stress-response system, led to the discovery of a novel epimerized RiPP called epipeptide (Benjdia et al., 2017c). Like proteusins, epipeptide biosynthesis is under the dependence of a radical SAM enzyme, EpeE (formerly called YydG) (Popp et al., 2021). The presence of two epimerizations in epipeptides (Figure 6A) proved to be essential to trigger cell envelop stress and membrane permeabilization (Popp et al., 2020). Interestingly, epipeptides are not restricted to *B. subtilis*, with epipeptide gene clusters also found in other bacterial species, including opportunistic pathogens from the human microbiota such as *Enterococcus faecalis*, *Streptococcus agalactiae* and *Staphylococcus epidermidis* (Benjdia et al., 2017a; Benjdia et al., 2017c; Popp et al., 2020; Popp et al., 2021). The bioactive form of epipeptides, purified from *B. subtilis* supernatant, showed that in contrast to proteusins, epipeptides possess the usual architecture of secreted bacterial peptides with a 32 amino-acid long leader peptide cleaved off during export. Similarly, proteusin epimerases share no significant homology with EpeE which harbors two [4Fe-4S] clusters, while proteusin epimerases contain only the radical SAM [4Fe-4S] cluster and a C-terminal RRE (Parent et al., 2018). Despite these structural differences, epipeptide and proteusin epimerases all appear to have leader peptide–independent activity and use a critical cysteine residue for catalysis (Benjdia et al., 2017c; Parent et al., 2018; Vagstad et al., 2019). Finally, the core peptide of proteusins such as PlpA with epimerizations occurring on Val and Ile residues perfectly aligns with the epimerized Val36 and Ile44 in epipeptide, questioning the evolution of these systems.

### Biological Functions of Proteusins and Epipeptides

The activity of proteusins remains largely unknown, with the noticeable exceptions of polytheonamide A which exerts a strong cytotoxic activity against mostly eukaryotic cells (Hamada et al., 2005) through membrane nano-channel formation (Iwamoto et al., 2010). The recently identified landornamide (Bosch et al., 2020) proved to exhibit a surprising antiviral activity although other RiPPs are known for their antiviral properties (Richard et al., 2015). Regarding epipeptides, they have been shown to inhibit Gram-positive bacteria (Benjdia et al., 2017c) through likely pore formation and affecting membrane fludity (Popp et al., 2020). Intriguingly, in the producing host, the LiaRS systems appear to be the main resistance mechanism (Popp et al., 2020).

### Mechanism of Radical SAM Epimerases

A mechanism for EpeE catalysis has been proposed based on isotopic labeling and mutagenesis experiments. After the reductive cleavage of SAM, it has been shown that EpeE catalyzes C_α_ H-atom abstraction on its target residues, Val36 and Ile44 (Figure 6B). The resulting C_α_-centered radical which losses its stereochemistry likely reacts with a cysteine residue from the radical SAM epimerase in order to conclude the epimerization reaction. Cys223 has been proposed to fulfill this critical role (Benjdia et al., 2017c), as its mutation led to peptide breakage consistent with the instability of C_α_-centered radical intermediates. This mechanism is reminiscent of a non-RiPP radical SAM enzyme, the spore photoproduct lyase (Chandor et al., 2006; Chandor-Proust et al., 2008; Benjdia, 2012), a DNA repair enzyme which also uses a protein cysteine residue as H-atom donor (Benjdia et al., 2012; Yang et al., 2013; Berteau and Benjdia, 2017). Similarly, sequence alignment of almost 70 PoyD homologs revealed a single and strictly conserved cysteine residue (Cys372) which was proposed to fulfill a similar role (Parent et al., 2018). This hypothesis was further supported by mutagenesis experiments showing that 1) *in vivo*, the C372A PoyD mutant failed to catalyze epimerization and 2) *in vitro*, it only catalyzes one epimerization event, in contrast to the wild-type enzyme (Parent et al., 2018). Collectively, these data support that radical SAM epimerases use a similar mechanism to alter peptide stereochemistry (Figure 6B). Once the thiyl radical is formed on the corresponding protein cysteine residue, it is unclear how this residue is regenerated for the next catalytic event. This last step is likely performed by an H-atom transfer pathway that remains to be identified (Parent et al., 2018). Solving the structures of radical SAM epimerases will help to determine if such H-atom transfer pathway is plausible and to confirm the role of these critical cysteine residues.

Interestingly, while in epipeptides, installation of epimerizations is independent events, in polytheonamide, it has been shown that *in vitro*, the first epimerization guides the second event which always occurs two residues away. Further studies investigating the influence of the core and leader sequence supported that the regioselectivity of radical SAM epimerases is also controlled by the core peptide and confirmed that these enzymes exhibit an extensive substrate promiscuity (Morinaka et al., 2014; Morinaka et al., 2017; Vagstad et al., 2019).

Importantly, radical SAM epimerases and sactisynthases appear to be a remarkable example of divergent evolution and diversification. While both family of radical SAM enzymes catalyze C_α_ H-atom abstraction, the fate of the radical intermediate differs depending on its reaction with a cysteine residue from the precursor peptide, leading to thioether bond formation, or from the protein itself, leading to epimerization.

### Carbon–Carbon, Carbon–Oxygen Bonds and Complex Rearrangements

The other group of RiPP-modifying radical SAM enzymes which has been intensively investigated are those catalyzing carbon–carbon and carbon–oxygen bond formation. While C–C and C–O bond formation catalyzed by various families of oxygenases including cytochrome P450 have been thoroughly characterized (Guengerich, 2018; Guengerich and Yoshimoto, 2018), anaerobic formation of such bonds has been only studied recently. In fact, the first member of this group of enzymes, PqqE, has been identified more than ten years ago (Wecksler et al., 2009; Haft and Basu, 2011). This enzyme, along with several other oxidases and proteases, is involved in the biosynthesis of pyrroloquinoline quinone (PQQ), a bacterial redox cofactor (Goosen et al., 1989).

**FIGURE 7:**
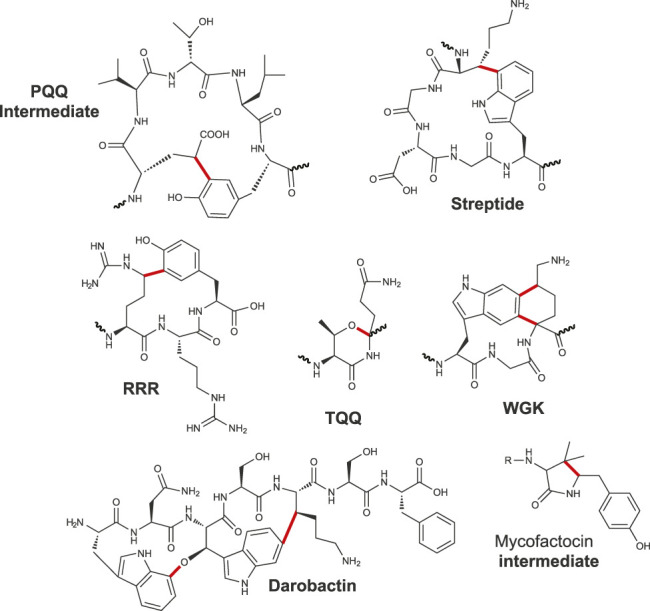
Carbon–carbon, carbon–oxygen bonds and complex rearrangement catalyzed by radical SAM enzymes.

Intriguingly, PqqE does not possess an RRE, in contrast to many RiPP-modifying enzymes, but instead requires another protein (PqqD) to serve as a *trans*-RRE. PqqD proved to be essential for the activity of PqqE on its precursor peptide (Barr et al., 2016) and the catalysis of a carbon–carbon bond formation between a glutamate (C_γ_) and tyrosine side chain (C3) (Figure 7). Once formed, this intermediate is further processed by PqqF/G/B and C to yield PQQ. While PQQ was known since more than forty years (Duine et al., 1980), existence of similar RiPPs containing crosslinks involving an aromatic residue has been revealed only recently. The second member of this group, called streptide, has been discovered while investigating quorum sensing in *Streptococcus thermophilus* (Ibrahim et al., 2007). Streptide is characterized by an unusual Lys (C_β_) to Trp (C7) bridge, and its expression is under the dependence of an Rgg regulator (Figure 7). Contrary to sactisynthases and radical SAM epimerases, the activity of the radical SAM enzyme catalyzing carbon–carbon bond formation in streptide is strictly dependent on the presence of the leader peptide (Schramma et al., 2015; Benjdia et al., 2017b). Following the characterization of streptide, other RiPPs from *Streptococci* were discovered sharing similar features (Figure 7). The RiPP WGK proved to contain a unique tetrahydro [5,6]benzindole cyclization motif derived from two C–C crosslinks between C5 (Trp)–C_α_ (Lys) and C6 (Trp)–C_δ_ (Lys) (Bushin et al., 2018). The RiPP TQQ was the first shown to contain, instead of a carbon–carbon bond, an ether crosslink between the side chain of a threonine residue and the C_α_ atom of a glutamine residue (Clark et al., 2019). The RiPP RRR exhibits an arginine residue (C_δ_ atom) linked to a tyrosine residue (C5 atom), a posttranslational modification strikingly similar to the one catalyzed by PqqE (Figure 7). For some of these RiPPs, the radical SAM enzymes appeared to have a relaxed specificity and a leader peptide–independent activity (Bushin et al., 2020), while others strictly require the leader peptide for activity (Benjdia et al., 2017b).

Interestingly, mining for antibiotics from the nematode microbiota, a novel RiPP called darobactin has been identified as harboring both a carbon–carbon bond, linking Trp to Lys like in streptide, and an ether bond linking two Trp residues (Imai et al., 2019). Surprisingly, here, the same radical SAM enzyme would catalyze the formation of both types of bonds, suggesting a commonality of mechanisms. However, while carbon–carbon bonds are likely formed by H-atom abstraction from the nonaromatic residue and subsequent addition of the carbon-centered radical to the phenyl or indole ring, formation of ether bonds is less well understood. Of note, formation of two different types of bonds has been previously reported for the radical SAM enzyme F_0_-synthase which has the unique ability to form C–C and C–N bonds during F_420_ biosynthesis (Decamps et al., 2012; Philmus et al., 2015). However, in sharp contrast to DarE involved in darobactin biosynthesis, F_0_-synthase possesses one radical SAM domain for each catalyzed bond.

Finally, mycofactocin is another remarkable example of RiPP in which one radical SAM enzyme catalyzes first an oxidative decarboxylation on a tyrosine residue followed by the formation of a carbon–carbon bridge with an adjacent valine residue (Figure 7) (Khaliullin et al., 2016; Khaliullin et al., 2017). Hence, like in darobactin (Imai et al., 2019), the mycofactocin biosynthetic pathway involves only one radical SAM enzyme catalyzing two distinct reactions. A similar complex rearrangement implying tyramine excision and formation of a carbon–carbon bond has been reported in spliceotide (Morinaka et al., 2018) (Figure 1). Further mechanistic studies will reveal how radical SAM enzymes perform such complex rearrangements, likely implying multi-catalytic cascades.

### Biological Functions

While several novel RiPP architectures have been uncovered in this group of RiPPs (Figure 7), we have still a very limited knowledge of their biological functions. Mycofactocin which shares striking similarities with PQQ has been recently demonstrated to be a novel RiPP-derived redox cofactor (Ayikpoe and Latham, 2019). Darobactin (Imai et al., 2019) by selectively targeting the essential BamA chaperone induces bacterial cell death (Imai et al., 2019). Several other RiPPs such as RRR, TQQ, WKG (Bushin et al., 2018; Caruso et al., 2019a; Caruso et al., 2019b; Clark et al., 2019), and streptide (Schramma et al., 2015) have their production tightly controlled by quorum-sensing system (Ibrahim et al., 2007), but their biological roles await further studies.

### Structures of Radical SAM Carbon–Carbon Cyclases

**FIGURE 8:**
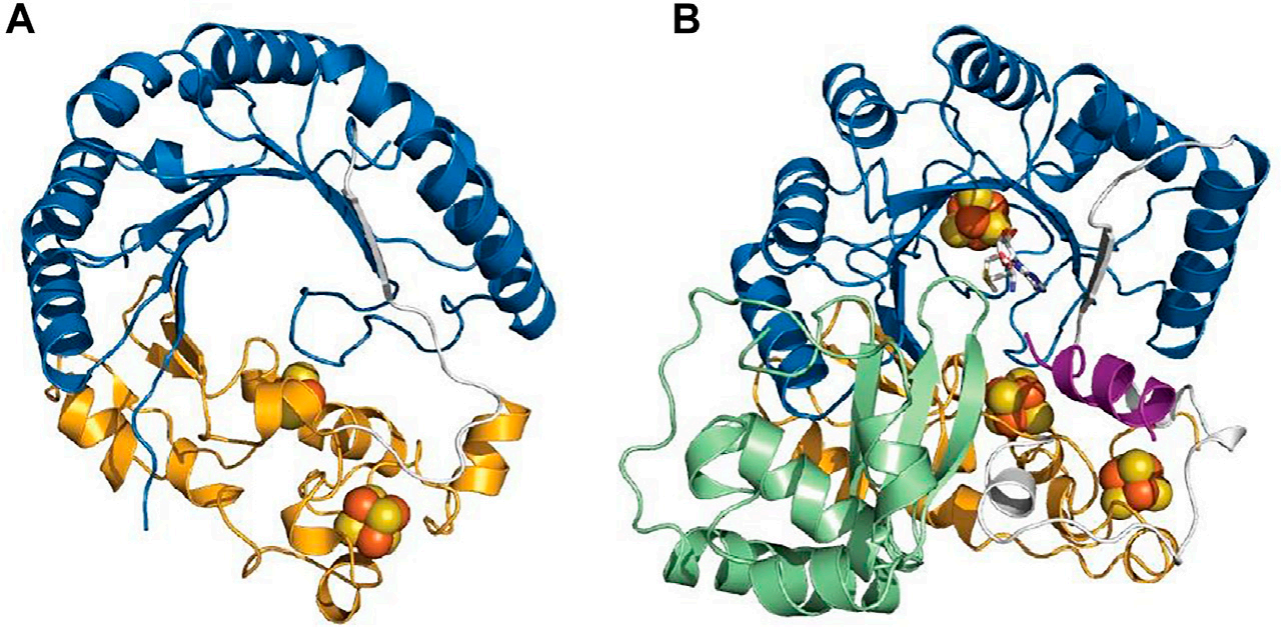
Structures of radical SAM enzymes catalyzing C–C bond formation. PqqE **(A)** and SuiB **(B)**. The radical SAM domain is depicted in blue, the N-terminal RRE domain is colored in green, and the C-terminal SPASM domain is shown in orange.

To date, only the structures of PqqE (Barr et al., 2018) and the radical SAM enzyme involved in streptide biosynthesis (SuiB) have been solved (Davis et al., 2017) (Figure 8). Like the ranthisynthase CteB, these enzymes share the canonical (β/α)_6_ TIM barrel fold but differ in the nature of auxiliary iron–sulfur clusters. Whereas SuiB harbors in its *C*-terminal part a SPASM domain characterized by two auxiliary [4Fe-4S] clusters, it has been shown that PqqE contains one [2Fe-2S] and one [4Fe-4S] auxiliary clusters, with possible conversion of the [2Fe-2S] into a [4Fe-4S] cluster (Zhu et al., 2020). In addition, one of the auxiliary clusters of PqqE is not coordinated by four cysteine residues, but by three cysteine residues and a conserved aspartate which likely modulates its redox properties (Barr et al., 2018; Tao et al., 2019).

While several studies have shown that these auxiliary clusters are not required for the homolytic cleavage of SAM, they are essential for the posttranslational modification to occur (Benjdia et al., 2016; Bruender and Bandarian, 2016; Bruender et al., 2016; Benjdia et al., 2017b). In contrast to sactisynthases and ranthisynthases, the auxiliary clusters from SuiB and PqqE are fully ligated by protein residues and thus cannot directly bind the substrate. Hence, a role as electron sinks has been proposed for these additional iron–sulfur clusters, similarly to anSME in which the two auxiliary [4Fe-4S] clusters are fully coordinated by cysteine residues (Benjdia et al., 2010; Goldman et al., 2013b).

Another salient feature of these RiPP-modifying enzymes is the presence of the RRE which exists either as a domain of the protein itself (SuiB) (Davis et al., 2017) or as a distinct protein partner (PqqD) (Evans et al., 2017; Barr et al., 2018). NMR studies of PqqD have shown that residues 20–34 of the peptide substrate PqqA likely bind in the cleft between β3-sheet and α3-helix (Evans et al., 2017). In addition, the structure of PqqD suggested a close binding between the PqqE active site and PqqD in order to get access to the core peptide and perform catalysis (Evans et al., 2017). Surprisingly, in SuiB, the leader sequence forms an α-helical structure and sits in the active site of the enzyme instead of being located in the RRE domain (Davis et al., 2017). Most interactions include H-bonds with the TIM barrel-SPASM domain bridging region, and a single peptide residue interacts with the RRE domain. Like for CteB, none of the modified residues were observed in the peptide-bound structure (Davis et al., 2017). Intriguingly, in the different structures solved, the position of the RRE relative to the radical SAM and SPASM/twitch domains differs (Figures 2A,B, 8), suggesting that instead of genuine variations, these orientations could represent diverse snapshots of substrate positioning movements (Grove et al., 2017; Barr et al., 2018; Grell et al., 2018). However, additional structures notably with substrate properly positioned in the active site will be required to explore this hypothesis.

## Outlook and Future Directions

In the last 5 years, significant advances in our understanding of radical SAM enzymes catalyzing RiPP modifications have been made. Notably, we have been witness of the discovery of novel posttranslational modifications (C_β_ and C_γ_ thioether bonds, ether bonds and epimerizations) and of novel reactions catalyzed by radical SAM enzymes (Bushin et al., 2018; Caruso et al., 2019a; Balty et al., 2019; Caruso et al., 2019b; Clark et al., 2019; Hudson et al., 2019; Balty et al., 2020; Bushin et al., 2020). With the first structures of RiPP-modifying enzymes solved including sactisynthase, ranthisynthase and carbon–carbon bond synthases, it is confirmed that the SPASM-domain present in all these enzymes fulfills diverse and probably combined roles (i.e., substrate binding and electron transfer pathway) (Davis et al., 2017; Grove et al., 2017; Grell et al., 2018). Regarding the RRE, it likely adopts diverse orientations with respect to the radical SAM domain, implying a possible motion of this domain for the correct positioning of the substrate (Grell et al., 2018). Only for SuiB, the leader peptide was located in the active site questioning the function of the RRE in this enzyme (Davis et al., 2017). Possible motions of the RRE might be a critical feature notably for enzymes installing multiple posttranslational modifications in RiPPs. However, a counterintuitive discovery of the last five years is that many radical SAM enzymes do not require the leader peptide to install posttranslational modifications at their correct location. Indeed, it has been demonstrated for several RiPPs, including subtilosin A (Benjdia et al., 2016), polytheonamide A (Parent et al., 2018), RRR (Caruso et al., 2019b), proteusins (Vagstad et al., 2019) and ruminococcin C (Balty et al., 2020), that the core peptide plays a major role to guide the installation of posttranslational modifications. Of note, in several instances, it has been shown that by introducing mutations in the core or the leader peptide, it is possible to change the selectivity of the RiPP modifying enzymes in order to generate designer RiPPs (Burkhart et al., 2017; Vagstad et al., 2019; Balty et al., 2020). Radical SAM enzymes and RiPPs are thus outstanding candidates to develop novel peptide-based antibiotics. Moreover, solving the structures of RiPP-modifying radical SAM enzymes with their peptide substrates properly positioned in their active site will not only give information on substrate recognition but also help to engineer novel RiPPs. Structural studies will be therefore instrumental to gain a deeper knowledge on these widespread biocatalysts.

Among radical SAM enzymes involved in RiPP biosynthesis, radical SAM methyl-transferases are attracting a growing interest (Montalban-Lopez et al., 2021). Indeed, they have been shown to use either cobalamin (vitamin B_12_) (Pierre et al., 2012; Blaszczyk et al., 2016) or SAM (Mahanta et al., 2017b; LaMattina et al., 2017) as ultimate methyl donor supporting a diversity of mechanisms and protein architectures. Additional biochemical and structural studies will be required to reveal the evolution forces that have shaped such a diversity of biocatalysts and fully understand their mechanisms.

Finally, while historically RiPPs have been mostly identified in bacteria isolated from the environment such as *Bacillus* species, the microbiome of simple eukaryotes (Freeman et al., 2012) (Imai et al., 2019) as well as the one of mammals and humans (Benjdia and Berteau, 2016; Benjdia et al., 2017a; Balty et al., 2020; Li and Rebuffat, 2020) have proved to be a rich source of novel chemistry and RiPPs (Table 1). As bacteria have to compete in this fierce and complex ecosystem for resources and ecological niches (Benjdia et al., 2011; Rothschild et al., 2018), it is hence not surprising that diverse commensal bacteria such as *Ruminococcus gnavus* (Balty et al., 2019), *Photorhabdus* (Imai et al., 2019), streptococci (Benjdia et al., 2017b; Bushin et al., 2018; Caruso et al., 2019b; Clark et al., 2019) and *Enterococcus* (Benjdia et al., 2017a; Benjdia et al., 2017c; Popp et al., 2020) have evolved a large diversity of effectors to gain a competitive advantage. RiPPs are also likely to play an underestimated role in bacteria communication and physiology (Chen et al., 2020; Li and Rebuffat, 2020). Indeed, many RiPPs have still unknown biological functions despite their regulation being tightly controlled notably by quorum sensing systems (Ibrahim et al., 2007). Further interdisciplinary studies will be required to decipher their biological role. It is most likely that the ongoing investigations of radical SAM enzymes will lead to the discovery of novel chemistry and reactions that will profoundly impact our understanding of the microbiome and provide novel opportunities for the development of innovative antibiotics.

**TABLE 1:** RiPPs produced by bacteria from diverse microbiome involving radical SAM enzymes (see Figures 1, 3, 6, 7 for corresponding structures).

RiPP	*Bacteria*	Posttranslational modification	Microbiome origin	Function	References
Darobactin	*Photorhabdus* sp	C–C and C–O bonds	Nematode	Antimicrobial	Imai et al. (2019)
Epipeptide	*Enterococcus faecalis, Streptococcus agalactiae, Staphylococcus epidermidis*	Epimerization	Human/mammalian	Antimicrobial	Benjdia et al. (2017c); Popp et al. (2020)
NxxcA	*Streptococcus orisratti* and *S*. *porci*	β-thioether cross-links	Mammalian	Unknown	Caruso et al. (2019a)
Polytheonamide	*Theonella swinhoei*	Epimerization and methylation	Marine sponge	Toxin	Hamada et al. (2005); Freeman et al. (2012); Parent et al. (2016); Parent et al. (2018)
Ruminococcin C	*Ruminococcus gnavus*	α-thioether cross-links	Human/mammalian	Antimicrobial	Balty et al. (2019); Chiumento et al. (2019); Balty et al. (2020)
SCIFF	*Clostridia*	β and γ thioether cross-links	Human/mammalian	Quorum sensing	Bruender et al. (2016); Precord et al. (2019); Chen et al. (2020)
Streptide	*Streptococcus thermophilus, S. mitis*	C–C bond	Human/mammalian	Unknown	Ibrahim et al. (2007); Schramma et al. (2015); Benjdia et al. (2017b)
Streptosactin (GGG)	*Streptococcus constellatus*, *Streptococcus gordonii*, *Streptococcus oralis* and *Streptococcus parasanguinis*	α-thioether cross-links	Human/mammalian	Antimicrobial	Bushin et al. (2020)
TQQ	*Streptococcus suis*	C–O bond	Mammalian	Unknown	Clark et al. (2019)
WGK	*Streptococcus equi, S. mutans*, and *S. ferus*	C–C bond	Human/mammalian	Unknown	Bushin et al. (2018)
